# RETRACTION: Effect of Laparoscopic and Open Distal Pancreatectomy on Postoperative Wound Complications in Patients With Pancreatic Cancer: A Meta‐Analysis

**DOI:** 10.1111/iwj.70398

**Published:** 2025-03-21

## Abstract

**RETRACTION:** HongC. and LiuW. , “Effect of Laparoscopic and Open Distal Pancreatectomy on Postoperative Wound Complications in Patients With Pancreatic Cancer: A Meta‐Analysis,” International Wound Journal 21, no. 2 (2024): e14564, 10.1111/iwj.14708.38351522 PMC10864682

The above article, published online on 13 February 2024, in Wiley Online Library (http://onlinelibrary.wiley.com/), has been retracted by agreement between the journal Editor in Chief, Professor Keith Harding; and John Wiley & Sons Ltd. Following an investigation by the publisher, all parties have concluded that this article was accepted solely on the basis of a compromised peer review process. The editors have therefore decided to retract the article. The authors did not respond to our notice regarding the retraction.

**RETRACTION:** C. Hong and W. Liu , “Effect of Laparoscopic and Open Distal Pancreatectomy on Postoperative Wound Complications in Patients With Pancreatic Cancer: A Meta‐Analysis,” International Wound Journal 21, no. 2 (2024): e14564, 10.1111/iwj.14708.38351522 PMC10864682

The above article, published online on 13 February 2024, in Wiley Online Library (http://onlinelibrary.wiley.com/), has been retracted by agreement between the journal Editor in Chief, Professor Keith Harding; and John Wiley & Sons Ltd. Following an investigation by the publisher, all parties have concluded that this article was accepted solely on the basis of a compromised peer review process. The editors have therefore decided to retract the article. The authors did not respond to our notice regarding the retraction.

